# Food intolerances in children and adolescents in Switzerland

**DOI:** 10.1007/s00431-022-04755-7

**Published:** 2022-12-13

**Authors:** Corinne Légeret, Clarissa Lohmann, Raoul I. Furlano, Henrik Köhler

**Affiliations:** 1 University Children’s Hospital Basel, Spitalstrasse 33, 4056 Basel, Switzerland; 2 Children’s Hospital Aarau, Tellstrasse 9, 5001 Aarau, Switzerland; 3 Faculty of Medicine, University of Basel, Basel, Switzerland

**Keywords:** Children, Intolerances, Food-avoidance

## Abstract

Adverse food reactions have become an important health concern in pediatrics. There are discrepancies between diagnosed and self-reported food allergies and intolerances, leading to food avoidance with possible psychological and nutritional implications in the latter. There is no data available so far on the number of children and adolescents, who reduce or completely avoid certain foods in their diet. The aim of this study was to determine the prevalence of children and adolescents in the Northwest of Switzerland, who omit foods from their diet because of a self-reported intolerance, as well as the prevalence of children and adolescents who eliminate well-tolerated foods for presumed health reasons. This is a cross-sectional study conducted in the Children’s hospitals of Aarau and Basel (Switzerland) and in 4 pediatric private practices. A total of 2036 children and adolescents (54% male and 46% female) between 1 month and 18 years (mean age: 7.4 years) were included, of which 316 (16%) participants reported to avoid foods due to intolerance. Lactose intolerance is the most frequent one. In 55% of all cases, no medical tests had been performed to confirm such an intolerance. Avoiding tolerated foods for presumed health reasons was stated by 251 (12%) participants.

*  Conclusions*: Clinicians should be aware of the high frequency of food avoidance in the pediatric population due to true intolerance, functional diseases, or due to lifestyle reasons. Milk is the most avoided food and half of the affected patients avoid foods without guidance by professional dieticians, therefore risking possible negative impacts on their physical and mental health. Pediatricians should perform further investigations or advise nutritional guidance if an avoidance of foods or specific diets occurs.

**Table:** 

**What is Known:** *• The prevalence of food intolerances in children and adolescents is unknown.* *• Eating behavior in children is influenced by the consumption of social media. *	
**What is New:** *• Based on this large cross-sectional study, it was shown that 16% of children and adolescents avoid foods, half of them without any medical advice.* *• Food avoidance is common among this Swiss cohort of children because of different reasons: lifestyle habits, functional diseases, true intolerances with possible consequences such as micronutrient deficiencies if not properly monitored.*

**Supplementary Information:**

The online version contains supplementary material available at 10.1007/s00431-022-04755-7.

## Background

Adverse food reaction is an umbrella term to describe an abnormal reaction to food or single food components and includes food allergy (FA), food intolerance, and hypersensitivities. FA is defined as an adverse health effect arising from a specific immune response occurring reproducibly on exposure to a given food [1] and can be classified according to the nature of the immune response as IgE-mediated, non-IgE-mediated, or mixed. Currently, the prevalence of food allergy in Europe is estimated to be 0.1–6% [2]. To confirm the diagnosis, a thorough history has to be taken, allergen-specific IgE may be measured and IgE sensitization can be confirmed by a skin prick test, but the gold standard remains the performance of a double-blind placebo-controlled food challenge [3]. The term food intolerance (FI) covers various non-allergic reactions to food. The underlying pathophysiology can be attributed to the reduced ability of the intestine to digest and absorb a certain food component, and a mismatch between supply and possible breakdown in the gut. The most common triggers are lactose and fructose, and classic symptoms include abdominal pain, bloating, diarrhea, and nausea [4]. The mechanisms of other FI such as nonceliac gluten- or wheat-hypersensitivity are not well understood, the symptoms can be similar to those of FA. Unfortunately, there is no single biomarker to confirm these diagnoses. Depending on the clinical history, different investigations may be performed, including blood, stool, or breath tests, food exclusion, and subsequent progressive food reintroduction. However, the gold standard remains the performance of a double-blind placebo-controlled food challenge [5].

The perception of food-induced symptoms is common among children and their parents in the general population but cannot always be objectified: Roehr et al. [6] performed a cross-sectional study of children and adolescents, of which 61% self-reported to have a FA. After clinical examination and performing food challenge tests, a FA could only be confirmed in 2.2% of all cases. Other studies did not only report large discrepancies between the prevalence of self-reported adverse reactions and the estimated prevalence of food allergy but also showed concomitant parental anxiety [7]. As clinical symptoms of food intolerances are unspecific, they can overlap with those of functional diseases, which are often treated by parents with special diets [8]. The resulting—mostly unnecessary—food avoidance can not only have a negative impact on psychological well-being but also serious nutritional implications. To make matters worse, special diets (gluten-free, lactose-free, low-carbohydrate diets, etc.) are advertised on social media by athletes, actors, models, etc., which has a demonstrable effect on teenagers’ own eating behavior [9, 10].

The aim of this study is to assess the current eating behavior among children in Switzerland. Specifically, to determine the prevalence of children and adolescents, who deliberately omit foods from their diet, as this may lead to micronutrient deficiencies.

## Methods

This is a cross-sectional study of pediatric patients between the age of 1 month and 18 years. It was performed in the Children’s Hospital of Aarau and Basel and in four different pediatric private practices in Switzerland between January and May 2022. Questionnaires were handed out in the waiting room of outpatient clinics and during registration in private practices by instructed medical staff only and standardized instructions to parents and children were given. Participation was voluntary and anonymous. Children filled out the questionnaire by themselves if they were 12 years or older, otherwise, the parents had to reply to the questionnaires. The aim was to obtain a representative picture of the eating behavior of children of all ages, so only few exclusion criteria were defined: a place of residence outside the Northwestern part of Switzerland recently arrived refugees, hospitalized oncological patients, and psychiatric patients, as well as critically ill outpatients in the emergency room.

### Questionnaire

The questionnaire had been initiated by the investigators and was available in German, English, and French and for the top ten languages, an interpreter was booked in 2022 in the Children’s Hospital, namely, Tigrinya, Albanian, Arabic, Farsi, Tamil, Dari, Turkish, Kurdish Sorani, Italian, and Portuguese. Only bilingual translators were involved and except for Tigrinya, Farsi, and Kurdish Sorani, an independent backward translation was performed to ensure the accuracy. A first version of the questionnaire was distributed to 14 patients (filled out by 8 teenagers and 6 parents) and tested for usefulness and clarity of the questions, which then were slightly modified based on the answers received. In the final questionnaire following questions were asked age, sex, place of birth of the child and the parents, population of residence (city, locality with > 10,000 inhabitants, rural locality, or village), and the level of education of both parents (less than 7 years of school, mandatory school, pre-apprenticeship, vocational apprenticeship or vocational school, high school, vocational baccalaureate or diploma school, higher technical and professional education, university or technical college and not determinable, unknown). Information about the underlying disease and known allergies was collected. Participants were specifically asked what food or food ingredients are eliminated from the diet because of an intolerance, how the diagnosis was made and by whom, and what symptoms occur if the food is ingested. A further question addressed whether foods or food ingredients, which do not provoke any symptoms, are reduced to increase health. Questions regarding the patient’s characteristics were closed, other questions were open with different suggestions (the full questionnaire is available as supplementary material).

### Definitions

Answers concerning age, sex, and the statement of the presence or absence of a food intolerance were mandatory. The term “food intolerance” regroups all adverse reactions to foods stated by the participants, which had not been diagnosed as allergies up to this time point. A specific food was interpreted either as causing a known allergy or as a self-reported food intolerance. “Known allergy” was defined as an allergy, previously diagnosed by a family doctor, a pediatrician, or an allergologist. Place of residence was defined as urban or rural (> 10,000 or < 10,000 inhabitants). The family background was classified as abroad if at least one parent was not born in Switzerland. The category abroad was then split into groups following a geographical classification: Central and Western Europe, Eastern and Southeastern Europe, Southern Europe, Middle East and Central Asia, South and Southeast Asia, Africa, and other regions. If the parents were born in different regions, we assigned it to the more distant region from Switzerland. Parent’s educational history: for this variable, the higher-educated parent was chosen, the category was divided into two groups (≤ high school and > high school).

### Statistical analysis

**Fig. 1:**
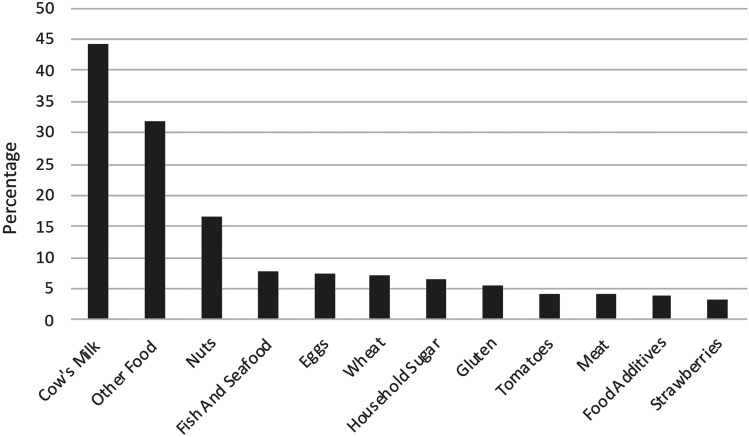
Avoided foods due to intolerance (*n* = 316). Cow’s milk includes lactose, milk, and milk protein; nuts: single, multiple, or all nut types; food additives: sorbit, xylit, sweeteners, and citric acid

**Fig. 2:**
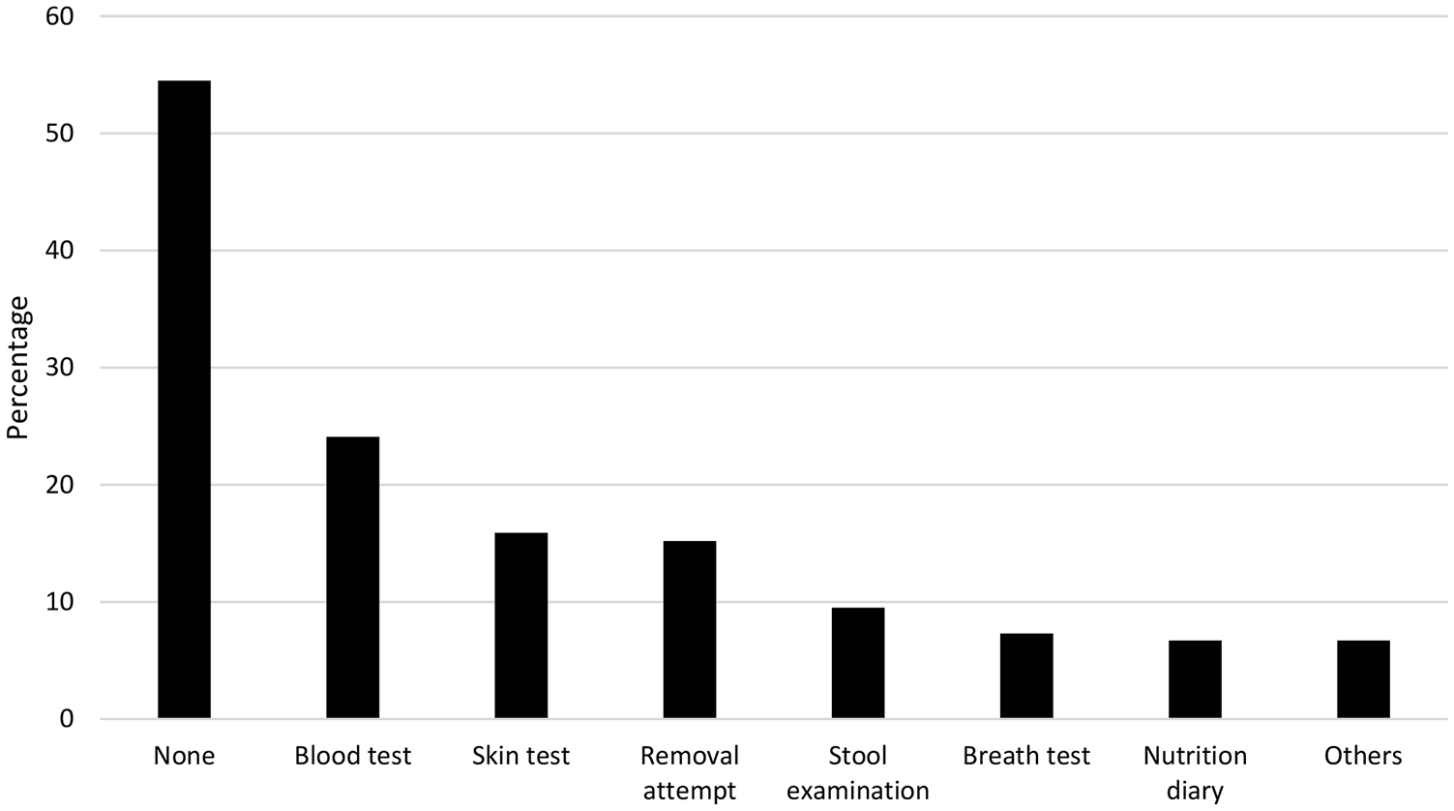
Tests performed for diagnosis of food intolerance. Conducted medical investigations (*n* = 315). Others: gastrointestinal endoscopy, alternative medicine investigations, genetic tests, ultrasound, physical examination, and unknown

**Fig. 3:**
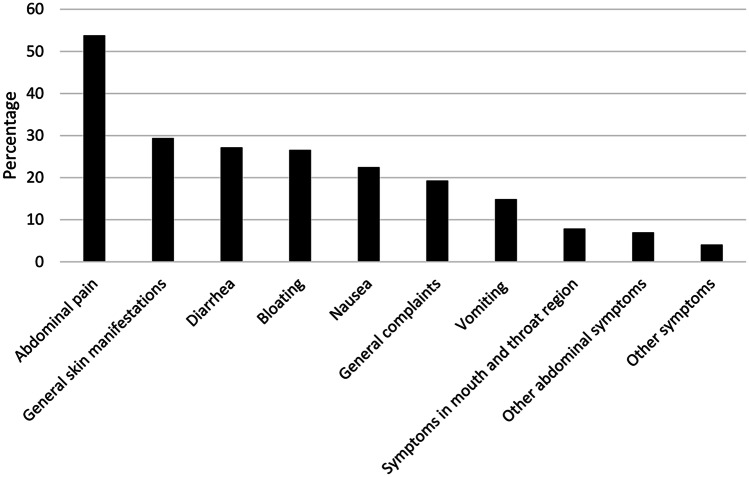
Reported symptoms by ingestion of suspected food (*n* = 315). General skin manifestations: rash, reddening, itching, and not specified; general complaints: headache, tiredness, concentration difficulties, performance reduction, restlessness/hyperactivity, shivering, apathy, and mood swings; symptoms in mouth and throat region: itching, swelling, rash, pain, aphthous ulcers, skin cracks, and alterations of the tongue; other abdominal symptoms: abdominal fullness, other changes in bowel habits, and regurgitation

Means with standard deviation (SD) were calculated for each of the measurements of interest, Shapiro–Wilk was applied to test normality. Univariate and multivariate analyses using *χ*2 test and logistic regression were applied with the exposure of all characteristic variables collected. Therefore, dependent variables were age, sex, place of residence (> 10,000 or < 10,000 inhabitants), highest education of parents, underlying diseases, and known allergies. *P*-values < 0.05 were considered statistically significant. All data were analyzed with RStudio 2022.02.2. Responses from the open-ended questions and categorical variables have been divided into subcategories (see section above) so that they could be analyzed quantitatively (see Figs. 1, 2, and 3).

### Ethical statement

The present study was conducted in accordance to the ethical principles laid down in the Declaration of Helsinki and its later amendments. Furthermore, it was approved by the local ethical committee (Ethics committee of Northwest Switzerland, EKNZ, trial number 2021–02,287).

## Results

The questionnaire was distributed to 2471 children and adolescents or their parents, of which 2042 participated (response rate of 83%). Six participants were excluded (4 were older than 18 years, one lived outside of Switzerland and one was a Ukrainian refugee). Hence, a total of 2036 questionnaires were finally included. Of the included questionnaires 153 (7.5%) were in another language than German (47 in English, 35 in French, 21 in Italian, 14 in Albanian, 11 in Turkish, 8 in Portuguese, 7 in Arabic, 6 in Tigrinya, 2 in Tamil, and 1 respectively in Farsi and Dari). In the Children’s Hospital of Aarau, 937 questionnaires have been filled out, 842 in the University Children’s Hospital of Basel, and 257 in pediatric private practices. Participants were 7.4 years old on average, for further characteristics we refer to Table 1.

**Table 1:** Characteristics of participants (*n* = 2036) and the occurrence of self-reported intolerances (SFI) and avoidance of tolerated food for health reasons (ATF)

	*N* total (%)	*SFI* (%)	*ATF* (%)
Age			
0–2 years old	438 (21.5)	57 (13)	52 (11.9)
3–5 years old	420 (20.6)	49 (11.7)	53 (12.6)
6–10 years old	564 (27.7)	86 (15.2)	73 (15.2)
11–14 years old	394 (19.4)	72 (18.3)	50 (12.7)
15–18 years old	220 (10.8)	52 (23.6)	23 (10.5)
Sex			
Male	1102 (54.1)	160 (14.5)	130 (11.8)
Female	934 (45.9)	156 (16.7)	121 (13)
Family background			
Swiss	883 (43.4)	138 (15.6)	109 (12.3)
Abroad	1085 (53.3)	165 (15.2)	137 (12.6)
Central and Western Europe	300 (14.7)	53 (17.7)	49 (16.3)
Eastern and Southeastern Europe	298 (14.6)	33 (11.1)	39 (13.1)
Middle East and Central Asia	109 (5.4)	16 (14.7)	4 (3.7)
Southern Europe	108 (5.3)	17 (15.7)	10 (9.3)
South, East, and Southeast Asia	87 (4.3)	20 (23)	7 (8)
Africa	85 (4.2)	9 (10.6)	11 (12.9)
Other (North, Middle, and South America, Northern Europe, Oceania)	98 (4.8)	17 (17.3)	17 (17.3)
Missing answer	68 (3.3)		
Highest education of parents*			
≤ College, vocational or intermediate diploma school*	786 (38.6)	101 (12.8)	76 (9.7)
> College, vocational or intermediate diploma school**	1157 (56.8)	199 (17.2)	164 (14.2)
Education status not stated or unknown	93 (4.6)		
*Less than 7 years of school, compulsory school, pre-apprenticeship, professional apprenticeship, professional school **Higher education course, higher professional education, university, technical college			
Place of residence			
Urban (> 10′000 inhabitants)	965 (47.4)	165 (17.1)	127 (13.2)
Rural (< 10′000 inhabitants)	1062 (52.2)	149 (14)	124 (11.7)
Missing answer	9 (0.4)		
Previous condition*			
Yes	311 (15.3)	74 (23.8)	55 (17.7)
Atopic disease	61 (3)	24 (39.3)	10 (16.4)
Disease of the nervous system	59 (2.9)	13 (22)	12 (20)
Gastroenterological disease	35 (1.7)	9 (25.7)	5 (14.3)
Genetic disease	25 (1.2)	6 (24)	5 (20)
Endocrinological	24 (1.2)	4 (16.7)	5 (20.9)
> 1 disease	17 (0.8)	5 (29.4)	5 (29)
Other disease (nephrological, cardiological, hemato-oncological, pneumological, rheumatological, musculoskeletal, metabolic, infectious, dermatological, unknown)	90 (4.4)	13 (14.4)	12 (13.3)
No	1700 (83.5)	238 (14)	195 (11.5)
Missing answer	25 (1.2)		
Known allergies**			
Yes	179 (8.8)	53 (29.7)	16 (8.9)
Pollen allergy	80 (4)	18 (18)	7 (8.8)
Mixed (≥ 2 allergies of different subgroups)	37 (1.8)	14 (37.8)	6 (16.2)
Food allergy	31 (1.5)	12 (38.7)	3 (9.7)
Other allergens (Dust mite, animal hair, unknown, bee venom, antibiotics)	31 (1.5)	9 (29)	0 (0)
No	1787 (87.8)	251 (14)	227 (12.7)
Missing answer	70 (3.4)		

^*^χ2 test: significant effect in both SFI and ATF, *p* < 0.01; **χ2 test: significant effect in SFI

To have a food intolerance was stated by 316 (16%) participants. Multiple logistic models revealed a statistically significant association between self-reported food intolerance (SFI) and increasing age (*p* < 0.01; odds ratio (OR) 1.04; confidence interval (CI) 1.02–1.07), an underlying atopic disease (*p* < 0.001; OR 3.26; CI 1.74–5.96), and a food allergy (*p* < 0.01; OR 3.67; CI 1.63–7.93), otherwise, no statistically significant association were found between SFI and participants’ characteristics (Table 2). Of the 316 participants with SFI, 30% (95/316) reported avoiding more than one food. Cow’s milk was the most avoided food (44%), followed by nuts (17%), fish and seafood (7.9%), and eggs (7.6%), see Fig. 1. The suspicion of the diagnosis was mostly based on observation by a family member (57%), in 34% of the cases by a pediatrician or a hospital doctor, in 20% by an alternative therapist (kinesiologist, traditional Chinese medicine, osteopath, etc.) and in 13% by a specialist (pediatric gastroenterologist, allergist, dermatologist, dietician), see supplementary figure. Further investigations had been performed: none in 55%, in 24% a blood test, in 16% a skin test, in 15% a removal attempt, in 9.5% a stool examination, in 7.3% a breath test, and 6.7% kept a food diary, see Fig. 2. Reported symptoms are shown in Fig. 3.

**Table 2:** Multivariable analysis for identifying potential relation between exposure variables and the outcome of self-reported food intolerances with separation of the variable family background abroad into subgroups

Characteristics	OR (95% CI)	*P*
Age	1.04 (1.02–1.07)	< 0.01
Female	1.26 (0.97–1.64)	0.09
Rural place of residence	0.84 (0.64–1.09)	0.19
Central and Western Europe	1.1 (0.76–1.59)	0.6
Eastern and Southeastern Europe	0.84 (0.54–1.28)	0.43
Middle East and Central Asia	0.89 (0.46–1.63)	0.73
Southern Europe	0.93 (0.49–1.65)	0.8
South, East, and Southeast Asia	0.93 (0.94–2.93)	0.07
Africa	0.6 (0.24–1.26)	0.21
Higher education	1.23 (0.93–1.64)	0.15
Previous Condition	1.54 (1.10–2.13)	< 0.01
Allergy	1.95 (1.31–2.85)	< 0.001
Atopic disease	3.26 (1.74–5.96)	< 0.001
Disease of the nervous system	1.5 (0.71–2.9)	0.25
Gastroenterological disease	1.18 (0.42–2.84)	0.73
Genetic disease	1.84 (0.66–4.49)	0.21
Endocrinological disease	1.24 (0.35–3.42)	0.71
> 1 disease	1.83 (0.5–5.4)	0.31
Pollen allergy	1.35 (0.72–2.39)	0.33
Mixed allergy	1.74 (0.75–3.79)	0.17
Food allergy	3.67 (1.63–7.93)	< 0.01

A total of 251 (12%) participants stated avoiding tolerated food for health reasons (ATF). There was a statistically significant correlation between ATF and higher education (*p* < 0.05; OR 1.47; CI 1.07–2.02), a family background in the Middle East and Central Asia (*p* < 0.05; OR 0.25; CI 0.06–0.69), an underlying disease of the nervous system (*p* < 0.01; OR 2.58; CI 1.25–5.00), and more than one underlying disease (*p* < 0.05; OR 3.85; CI 1.18–11.09), otherwise no statistically significant correlations were found between ATF and participants’ characteristics, see Table 3. The most commonly avoided food component was sugar (incl. soft drinks and sweets) in 45% (112/251), animal products (meat, fish, egg, milk products) in 32% (79/251), wheat and/or gluten in 22% (56/251), fatty foods in 6% (15/251), carbohydrates in 5.6% (14/251), and other foods (processed foods, white flour, caffeine, additives, salt) in 19% (48/251), see supplementary Figure.

**Table 3:** Multivariable analysis for identifying potential association between exposure variables and the outcome of avoidance of tolerated foods with separation of the variable family background abroad into subgroups

Characteristics	OR (95% CI)	*P*
Age	1 (0.97–1.03)	0.9
Female	1.04 (0.78–1.38)	0.79
Rural place of residence	0.99 (0.74–1.32)	0.93
Central and Western Europe	1.22 (0.82–1.79)	0.32
Eastern and Southeastern Europe	1.3 (0.84–1.98)	0.22
Middle East and Central Asia	0.25 (0.06–0.69)	< 0.05
Southern Europe	0.85 (0.4–1.64)	0.66
South, East and Southeast Asia	0.63 (0.24–1.39)	0.3
Africa	1.21 (0.54–2.44)	0.62
Higher education	1.47 (1.07–2.02)	< 0.05
Previous Condition	1.54 (1.10–2.13)	< 0.001
Atopic disease	1.91 (0.84–3.93)	0.1
Disease of the nervous system	2.58 (1.25–5.00)	< 0.01
Gastroenterological disease	1.32 (0.38–3.53)	0.62
Genetic disease	1.88 (0.61–4.82)	0.22
Endocrinological disease	2.1 (0.74–6.25)	0.11
> 1 disease	3.85 (1.18–11.09)	< 0.05
Allergy	0.6 (0.33–1.04)	0.09

## Discussion

In our Swiss cohort of over 2000 children and adolescents, 16% of children or their parents reported to avoid foods due to perceived intolerance. The literature in regard to the prevalence of intolerances in children is sparse because it can be hard to diagnose: while breath tests can be used to make the diagnosis of lactose or fructose intolerance, many other intolerances (e.g., non-coeliac gluten hypersensitivity) lack specific diagnostic markers or procedures. Often, they rely purely on subjective observations and therefore the diagnosis may be challenging, especially since patients often present with unspecific symptoms, such as abdominal pain and bloating. The diagnosis of a food allergy, in contrast, is more specific and can be better objectified.

A European-wide study of children between 7 and 10 years of age showed a prevalence of self-reported food allergies (FA) ranging from 13 to 46%. This variation seems to depend on the geographical region [11]: a study in Germany showed a prevalence of 38% of reported adverse food reactions while a study in the UK reported that a total of 12% of 11-year-old and 12% of 15-year-old have a problem related to food [6, 12]. A lower prevalence of food hypersensitivity has been reported in Southern European countries, as well as in Turkey. This may be due to genetic, cultural, or dietary factors, as well as public awareness [13, 14]. Evidence shows that the percentage of the adverse food reactions, which are diagnosed by a double-blind placebo-controlled food challenge, is up to 15 times lower. These results give us an impression on the number of people changing their dietary habits due to perceived adverse food reactions [2].

In this study, cow’s milk (mainly lactose) was the most common self-reported food-causing symptoms, which is in line with the results of other studies. There is still an ongoing confusion in the general population between cow’s milk allergy (CMA) and lactose intolerance: mostly children under the age of 2 years are affected by a CMA, whereas it is unlikely in school-aged and older children and adolescents [11, 15]. After weaning, lactase levels decline in 70% of the world’s population, which is the physiological basis of lactose intolerance, a carbohydrate malabsorption, but rarely appears before the age of 5 years [16].

The association between self-reported lactose intolerance, objective findings of tests, and clinical outcome of dietary intervention is variable and often overestimated [17, 18]. This is a common problem, as symptoms of intolerances can be subtle and may overlap with those of functional diseases. Children with functional abdominal problems may benefit of a FODMAP diet [19]: an acronym for fermentable oligosaccharides, disaccharides, monosaccharides, and polyols, which can increase osmotic activity, followed by water secretion into the lumen of the small intestine. Fermentation by gut bacteria stimulates colonic gas production—all processes are associated with the pathophysiology of functional abdominal pain [20]. In children following a FODMAP diet, lactose is advised to be completely avoided. In this study, most children avoided lactose without further diagnostic procedures, therefore it remains unclear, whether they do have a lactose intolerance or if they suffer from a functional disease.

The increasing prevalence of SFI with age might be associated with the increasing prevalence of lactose intolerance in later childhood and adolescence [21] or with the fact, that the diet of adolescents is strongly influenced by social media [22, 23]. In social media, the number of accounts promoting a “healthy lifestyle” (diets and physical workout) has been increasing in the last decade [24]. Its content is unfortunately not created by professional dieticians, therefore nutritional advises are questionable, [25]. The consumption of health-related content on social media shows a direct effect on the eating behavior of—mainly—girls [26].

There seems to be a higher prevalence of SFI in families with higher education [27], but without statistical significance in the multivariable model. The higher prevalence of SFI in participants with known food allergies and/or atopic diseases is explained by the fact that these parents are more attentive to a possible reaction to food and might be more fearful [28].

The economist forecasted that “2019 will be the year that veganism goes mainstream” [29], as it combines a more ethical and environmental approach to life, meeting the current lifestyle in developed countries [30]. As a balanced, healthy vegan diet is more expensive than a freshly cooked standard omnivore diet [31], living this lifestyle cannot be afforded by everyone, which might explain that a higher education was statistically significant in our multivariable analyses concerning ATF. In our study around 3% reported to be on a vegetarian or vegan diet. Gluten-free diet has become more popular in non-coeliac patients for supposed health benefits [32], as seen in our cohort. The higher prevalence of ATF in participants with an underlying disease of the nervous system might be caused by rumors that gluten- and casein-free diet improves autism and a restriction of sweeteners and colorants and preservatives ADHD [33–35].

In our survey, more than 50% of the participants, who reported an intolerance, avoided the potential trigger food(s) of their own initiative and without further medical investigation and therefore without medical support. An appropriate professional guidance is important to avoid over-restriction of a diet, to regularly reassess tolerance [36], and prevent deficiencies of micronutrients, which are important for growing children. Dairy foods contain essential nutrients (protein, magnesium, potassium, zinc, and vitamin D) and are the main source for calcium intake, which is crucial to secure bone health [37].

Furthermore, in patients with lactose intolerance (including self-diagnosed) additional intolerance to other products, especially to those that can lead to bloating (raw legumes and dried fruits) is often a problem, which leads to a higher risk of a restrictive diet negatively impacting health [17]. A gluten-free diet is associated with a higher intake of saturated fat, while the intake of fiber and micronutrients (e.g., iron, folate, zinc) is decreased [38]. A nutritionally adequate lacto-ovo-vegetarian diet is feasible, a vegan diet, especially in younger children should be controlled by specialists to prevent deficiencies since food from animal sources contain essential nutrients (protein of high biological value, calcium, iron, iodine, selenium, zinc, vitamin A, D, B2, and B12) that are not easy to compensate in a strict vegan diet [39, 40].

This is the first study in Switzerland to assess the prevalence of food avoidance in the pediatric population, providing an impression of the current situation in regard to the changing eating behavior in central Europe. Questionnaires in different languages made participation accessible to a wide range of families and therefore the response rate was high. The main limitation of the study is that the data is solely based on participants’ subjective accounts. Without further investigations, we cannot differentiate food intolerances from possible functional diseases. To avoid confusion in the participants, it was specifically asked for food allergies and food intolerances. However, it can also be judged to be a strength of this study, to provide real-world data of the actual eating behavior of children and adolescents. Another limitation of this study is that the questionnaire has not been validated; however, it was pilot-tested.

## Conclusion

In this cohort of over 2000 Swiss children and adolescents, 16% reported to avoid foods due to intolerance, an additional 12% avoid certain foods to increase their health. More than half of the participants reporting intolerance did not have medical assistance in making the diagnosis. Although the underlying cause (intolerance, functional disease, lifestyle) remains unclear, it demonstrates that the number of children and adolescents avoiding foods without guidance and the possible negative impact on health should not be underestimated. Awareness on this matter must be raised in pediatricians to ensure that avoidance of foods and specific diets are directly addressed and further investigations or nutritional guidance is advised.

## Supplementary Information

Below is the link to the electronic supplementary material.

Supplementary file1 (DOCX 17 KB)

Supplementary file2 (DOCX 17 KB)

Supplementary file3 (PDF 996 KB)

## Abbreviations

ATF: Avoidance of tolerated food for health reasons
FA: Food allergy
FI: Food intolerance
SFI: Self-reported food intolerance
