# Supplementary material for: Food intolerances in children and adolescents in Switzerland
Source: Eur J Pediatr. 2022 Dec 13;182(2):867–75. doi: 10.1007/s00431-022-04755-7 (PMC9899185; doi:10.1007/s00431-022-04755-7)
Supplement: Supplementary file 1 — Supplementary file1 (DOCX 17 KB) [file 431_2022_4755_MOESM1_ESM.docx]

Additional Figure) Who did initially suspect a food intolerance?

n= 315; Alternative medicine staff: Naturopath, kinesiologist, traditional Chinese medicine
