# Supplementary material for: Food intolerances in children and adolescents in Switzerland
Source: Eur J Pediatr. 2022 Dec 13;182(2):867–75. doi: 10.1007/s00431-022-04755-7 (PMC9899185; doi:10.1007/s00431-022-04755-7)
Supplement: Supplementary file 2 — Supplementary file2 (DOCX 17 KB) [file 431_2022_4755_MOESM2_ESM.docx]

Additional Figure) Avoidance of tolerated food for health reasons (n=251)

Animal products: meat, fish, eggs, milk products, lactose; others: processed foods, white flour, caffeine, food additives, salt, unknown; carbohydrates: including specific foods like bread or pasta
