# Supplementary material for: Food intolerances in children and adolescents in Switzerland
Source: Eur J Pediatr. 2022 Dec 13;182(2):867–75. doi: 10.1007/s00431-022-04755-7 (PMC9899185; doi:10.1007/s00431-022-04755-7)
Supplement: Supplementary file 3 — Supplementary file3 (PDF 996 KB) [file 431_2022_4755_MOESM3_ESM.pdf]

Food intolerances in children and adolescents

Participants' questionnaire

With this survey, we would like to assess the frequency of food intolerances among children and adolescents in Northwestern Switzerland. All data will be treated confidentially and are used anonymously. Participation is voluntary and consent can be withdrawn any time. We recommend that children under 12 years of age complete the questionnaire together with a parent.

☐ I agree that the data provided may be used anonymously for research purposes.

If you do not want to participate, you can specify the reason here:

☐ Language comprehension problems
☐ My child has no food intolerances.
☐ Other: ………………………………

This questionnaire was filled out:

☐ At the Children's Hospital Aarau
☐ At the UKBB
☐ In a pediatric practice

The questionnaire was filled out by: ☐ Mother ☐ Father ☐ Child with a parent ☐ Child or teenager alone

1. Age of the child in years: ………………………

2. Sex of the child: M ☐ F ☐

3. Place of birth of the child: ……………………………………….

3.1. Place of birth of the mother: ……………………………………

3.2. Place of birth of the father: …………………………………….

4. Current place of residence of the child/adolescent:

☐ City (Aarau, Baden, Basel-Stadt, Olten, Liestal)
☐ Rather urban (locality > 10'000 inhabitants)
☐ Rather rural
☐ Village

5. Does your child have an underlying disease? NO ☐ ☐ Yes, the following: …………………………………

6. Does your child have allergies, which have been diagnosed by a family docter, a paediatrician or an allergologist and require treatment? ☐ No ☐ Yes, the following:

……

7. What is your highest completed education?

Mother	Father	
☐	☐	Less than 7 years of school
☐	☐	Mandatory school
☐	☐	Apprenticeship
☐	☐	Vocational apprenticeship, vocational school
☐	☐	High school, vocational baccalaureate, diploma school
☐	☐	Higher technical and professional education
☐	☐	University, technical college
☐	☐	Not determinable, unknown

---

*The following questions apply to **your child** or **to you (child or adolescent).***

8. Does your child/do you have a food intolerance, or are there food groups that are not good for your child/you: ☐ Yes ☐ No

- If yes, what foods/food ingredients are you trying to avoid with your child/are you trying to avoid?
  ☐ Lactose ☐ Fructose (fruit sugar) ☐ Sucrose (household sugar)
  ☐ Sorbitol ☐ Histamine ☐ Gluten ☐ Wheat ☐ Eggs ☐ Nuts
  ☐ Fish and seafood ☐ Meat
  ☐ Others: ……………………………………………………………………………………………

- Who suggested that your child/you has/have these food intolerances?
  ☐ Own/parental observation
  ☐ Pediatrician ☐ Gastrointestinal specialist ☐ Hospital doctor
  ☐ Naturopath ☐ Kinesiologist ☐ Parents counselling
  ☐ Relatives ☐ Friends ☐ Internet research
  ☐ Others: ……………………………………………………………………………………………

- Have medical examinations been made?
  ☐ No ☐ Blood test ☐ Stool examination ☐ Breath test
  ☐ Gastroscopy/colonoscopy ☐ Skin test ☐ Removal attempt
  ☐ Food diary ☐ Genetic test
  ☐ Others: ……………………………………………………………………………………………

- What symptoms occur when your child/you consume the food(s) in question?

☐ Abdominal pain ☐ Nausea ☐ Diarrhea ☐ Headache
☐ Flatulence ☐ Skin problems ☐ Tiredness ☐ Bloating
☐ Concentration difficulties ☐ Vomiting ☐ Performance reduction
☐ Others: ……………………………………………………………

In case of multiple intolerances, please specify which food the complaints refer to:
…….

9. Are there foods (e.g., gluten, wheat) that you are trying to reduce for health reasons, even if your child/you doesn't/don't get symptoms from them?

☐ No ☐ Yes, the following: ……………………………………………………………………
